# Survey on Mental Health Status in Iranian Population Aged 15 and Above One Year after the Outbreak of COVID-19 Disease: A Population-Based Study

**DOI:** 10.34172/aim.2022.35

**Published:** 2022-04-01

**Authors:** Ahmad Ali Noorbala, Azam Maleki, Seyed Abbas Bagheri Yazdi, Elham Faghihzadeh, Zarrintag Hoseinzadeh, Marzieh Hajibabaei, Seyedeh Elham Sharafi, Koorosh Kamali

**Affiliations:** ^1^Psychosomatic Medicine Research Center, Tehran University of Medical Sciences, Tehran, Iran; ^2^Social Determinants of Health Research Center, Zanjan University of Medical Sciences, Zanjan, Iran; ^3^Department of Mental Health, Ministry of Health and Medical Education of Iran, Tehran, Iran; ^4^Department of Epidemiology and Biostatistics, School of Medicine, Zanjan University of Medical Sciences, Zanjan, Iran

**Keywords:** COVID-19, General population, Iran, Mental health

## Abstract

**Background:**

Mental disorders are the most common health problems that affect different population groups. According to the national survey in 2015 based on General Health Questionnaire-28 (GHQ-28), 23.44% of Iranians older than 15 years were suspected of having a mental disorder. The study aimed to determine the mental health status of the population over 15 years of age in the Islamic Republic of Iran, one year after the outbreak of COVID-19 in 2020.

**Methods:**

The population-based study was performed on 24584 individuals over 15 years of age in Iran between December and February, 2020. The GHQ-28 was completed through telephone interviews. Data were analyzed using descriptive statistics, chi-square test, independent *t*-test, and multiple logistic regression at 95% confidence level.

**Results:**

The results showed that the mean age of participants was 44.18±16.47 years. The prevalence of mental disorders was 29.7%. Mental disorder was associated with female gender (OR=1.195, 95% CI 1.10–1.29), 25–44 years (OR=1.206, 95% CI 1.06–1.36), urban life (OR=1.116, 95% CI 1.04–1.19), illiteracy (OR=1.286, 95% CI 1.11–1.48), being divorced (OR=1.924, 95% CI 1.50– 2.45), and unemployment (OR=1.657, 95% CI 1.40–1.94). Among the participants and their families, 14.7% and 32.3% were infected with the disease, respectively. The COVID-19 mortality rate in their families was 13.2%. The prevalence of mental disorders in infected people (40% vs. 27.3%) and bereaved families (39.6% vs. 35.3%) was more than the non-infected groups.

**Conclusion:**

Our results showed that in Iran, the mental health of the general population had a rising trend compared to 2015, especially in people infected with COVID-19 and bereaved families. The observed difference may be due to the prevalence of the COVID-19 epidemic and rapid demographic, social, and economic changes in Iran. Planning to improve mental health in the mentioned population should be considered for the post COVID-19 era.

## Introduction

 According to the Charter of the World Health Organization (WHO), health is not only lack of illness or disability, but also refers to the “optimal state of physical, mental, and social well-being.” According to the WHO, the concept of mental health goes beyond lack of mental disorders and includes mental well-being, self-efficacy perception, independence and autonomy, merit and competence, intergenerational dependence, and self-fulfillment of potential mental and emotional abilities. On the other hand, behavioral-psychological disorder is a clinical condition that is accompanied by changes in certain thoughts, moods, emotions, or behaviors, as well as personal discomfort and anxiety or inefficiency of life. These changes are not in line with social norms and are abnormal, pathological, persistent, or recurrent.^1^ Mental health is closely related to public health and social factors affecting health; therefore, without mental health, access to other aspects of health is not possible. However, most mental disorders are preventable. Pregnancy and childhood interventions and care, life skills training, parenting, and marriage are among the most effective interventions to prevent mental disorders.^2^ Today, we are facing an alarming increase in the incidence of mental disorders around the world, which can lead to political turmoil, a wave of constant violence, and frequent changes in the social context of countries such that mental disorders and substance abuse are the most important causes of health problems in the world.^3^ Research has shown that psychiatric disorders are one of the most important components of the overall burden of diseases. In this regard, the findings of the Global Burden of Diseases Study (GBD 2015) have shown that psychiatric disorders are one of the main causes of the global burden of diseases.^4^ Of these patients, 75% live in low- and middle-income countries.^5^ Mental disorders are one of the most common health problems affecting the adult population. The GBD (2015) has estimated that seven of the 25 main causes of “years lived with disability” worldwide are related to mental disorders. Major depressive disorder is in the second place, and anxiety is in the ninth place.^6^

 Various studies have been conducted on the prevalence of mental disorders in Iran. In this regard, a systematic study was conducted on the prevalence of psychiatric disorders in Iran in 2007. In the mentioned systematic review, 35 studies were reviewed by the end of 2006, and the results showed that the prevalence of psychiatric disorders was 28.70% in screening studies and 18.60% in studies carried out using diagnostic interviews.^7^ In a study by Moradpour et al, that analyzed the results of the national mental health study in 2011 using the Bayesian method, the prevalence of psychiatric disorders in Iran was reported to be between 3.6% and 62.6%.^8^ In the first large-scale study on the epidemiology of mental disorders in Iran using the general health questionnaire-28 (GHQ-28), Noorbala et al showed that the prevalence of suspected mental disorders in a statistical sample of 35,014 individuals aged 15 and older was about 21% (25.9% women and 14.9% men).^9^ In another large-scale study conducted by Noorbala et al on a sample size of 36 000 individuals to assess mental health in the population over 15 years of age in urban and rural areas of Iran, the findings showed that 23.44% were suspected of having a mental disorder. The prevalence of mental disorders was 24.55% in urban and 20.89% in rural areas. Also, the prevalence of mental disorders increased significantly with age and was more common among women, divorced individuals, widows, and illiterate and low-literate, unemployed, and disabled individuals.^10^

 The coronavirus pandemic has been transmitted from human to human since late December 2019, which is not only a threat to the physical health of the community but has also caused confusion and uncertainty in the community due to the ambiguity of the mutant virus function.^11^ This disease has caused unbearable psychological pressures such as stress, anxiety, depression, and unresolved grief, and also the effects of post-traumatic stress have occurred in individuals with this disease. This issue has caused heavy damages for the affected communities.^12^ There is a variety of factors that may damage mental health and lead to increased anxiety and distress during the pandemic, such as personal concerns about the risk of infection and worries about other individuals’ health, financial and work problems, school closure, and reduced social ties. The COVID-19 crisis has made mental health services more difficult, and this is particularly worrying because individuals with underlying mental health backgrounds may be more vulnerable.^13^ The long-term effects of the COVID-19 crisis on mental health are currently unknown. There is evidence that major stressful events, such as natural disasters, can have lasting effects on mental health.^13^ In a retrospective study carried out in the United States, it was found that 18.1% of individuals who recovered from COVID-19 had one of the symptoms of psychiatric disorders within 14 to 90 days after the infection, 5.8% of whom were new cases of the disease. In individuals over 65 years of age, the prevalence of dementia increased by 1.6%.^14^ A cohort study conducted in China by Huang et al on patients with COVID-19 who had been discharged from the hospital between January 7 and May 29, 2020, showed that during 6 months after acute infection, COVID-19 survivors mainly experienced fatigue or muscle weakness, sleep problems, and anxiety or depression (anxiety or depression in 23% of patients).^15^ In another study, Shahriarirad et al showed that during the outbreak of COVID-19, 15.1% and 20.1% of the Iranian general population over 15 years of age had experienced clinical symptoms of depression and anxiety, respectively.^16^ Given the above-mentioned materials and the fact that mental disorders can cause years of severe disability, and considering that mental illnesses are among the most common diseases in the present age, with a brief look at the trend of mental and behavioral diseases and the problems caused by them in the world, paying serious attention to the diagnosis, care, and follow-up of mental health indicators is necessary. Therefore, the present research was conducted aiming to study the mental health status of the population over 15 years of age in the Islamic Republic of Iran, one year after the outbreak of COVID-19 (in 2020) to achieve the latest status of mental disorders in Iran and also investigate the relationship between mental disorders and the COVID-19 pandemic.

## Materials and Methods

 The national survey was conducted in the form of a population-based study aiming to determine the mental health status of individuals over 15 years of age in the Islamic Republic of Iran between December and February 2020. The study population in the present research was the population over 15 years of age living in Iran. Assuming the prevalence of psychiatric disorders at 30% (*P* = 0.3), the first type error at 0.05, the accepted error at 0.04, and the effect of cluster sampling equal to 1.6, the sample size for each province was calculated as 825 individuals. Considering 31 provinces, the total sample size was 25 575 individuals.

 Given the high penetration of landline and cell phones throughout the country and considering the lack of necessary and standard conditions for face-to-face surveys due to the prevalence of COVID-19, in the present study, the multi-stage random sampling method was used for data collection. Each province was considered as a cluster; in each province by observing the age and gender ratios, the random digit dialing sampling method was performed by trained questioners of the Jahad-Daneshgahi Polling Agency to access individuals. First, the number of samples for each province was determined according to the census and the statistical population of the study. Then, using a completely random method, landline and cell phone numbers were generated for each province using the codes specified for each provincial capital city. After generating landline and cell phone numbers (including all operators) for each provincial capital city (and affiliated villages), the interviewers randomly completed the questionnaires by observing the age and gender ratios. About two-thirds of the samples were assigned to cell phones, and the others were assigned to landlines. In landline calls, individuals living in residential houses were included in the statistical population of this survey, and commercial centers and public places (hospitals, schools) were considered guests and non-Iranian individuals outside the statistical population, and their questionnaires were not completed. The interviews were conducted by telephone, mostly in Persian, and in some provinces in Kurdish and in Azeri. The response rate in this survey was about 65%. The monitoring method was simultaneous, in such a way that the supervisors simultaneously monitored the interviewers’ method of performing interviews and warned the interviewer if an error occurred; in cases where more serious errors occurred, such as not asking complete questions or completing a questionnaire outside the statistical population, the mentioned questionnaire was removed and replaced with another sample.

###  Data Collection Method

 Data were collected using telephone interviews in two sections: Demographic characteristics and mental health status. The demographic characteristics questionnaire included age, gender, marital status, employment, place of residence, COVID-19 status in the individual, infection or death due to COVID-19 disease in relatives, and duration and severity of COVID-19 disease.

 To screen the individuals’ mental health status, the GHQ-28 was used. This questionnaire designed by Goldberg and Hillier (1979) includes 4 subscales: Somatization symptoms (questions 1–7), anxiety symptoms - sleep disorder (questions 8–14), social dysfunction (questions 15–21), and depressive symptoms (questions 22–28). Scoring is based on a 4-point Likert scale ranging from 1 (at all) to 4 (much higher than usual). The scoring method used in this study was the traditional scoring method in which the options were scored as (0-0-1-1) and the subject’s score varied between zero and 28. In this study, obtaining scores higher than 6 out of the total scores and those higher than 2 in the subscales indicated pathological symptoms. The validity and reliability of the Persian version of the questionnaire were reviewed and confirmed by Noorbala et al.^17^

###  Analysis of Results

 Data was weighted by the population covered for each province for estimating the cluster sampling. Weighted data were analyzed using the SPSS 16 software with descriptive statistics (number, percentage, mean, and standard deviation), chi-square test, independent *t* test, and multiple logistic regression with the inter method at 95% confidence level. To calculate the national prevalence of mental disorders, weighting was performed according to the population of each province. We tried generalized estimating equations (GEE) with an unstructured working correlation matrix and it showed that there were no correlations within 31 provinces. Therefore, the result of GEE was the same as logistic regression.

## Results

 A total of 25 200 questionnaires were completed. Six hundred and sixteen questionnaires were not analyzed due to incomplete data; therefore, the reported results included 24 584 samples.

 The results showed that the mean age of participants was 44.18 ± 16.47 years. The highest percentage of participants were city residents (72.65%), male (50.07%), in the age group 25 to 44 years (37.61%), employed (40.32%), and married (73.36%), and had education levels of above diploma & bachelor (26.38%). The highest percentage of participants’ residential homeownership was personal (79.5%). The overall prevalence of suspected mental disorders in the population over 15 years of age was 29.7%. Based on demographic variables, the percentage of suspected mental disorders in women (31.75%), city residents (29.49%), the age group 25 to 44 years (31.40%), and divorced (45.70%), illiterate (30.77%), and unemployment individuals (38.86%) was higher than other groups (Table 1).

**Table 1:** Mental Disorders Suspicion Rate One-Year Post-COVID-19 (2020)

**Demographic Factors**		**Sample size**		**Suspected of Mental Disorders**		
		**No.**	**%**	**No.**	**%**	**95% Confidence Interval**
Gender	Male	12 309	50.07%	3195	26.64%	(25.86–27.44)
	Female	12 275	49.93%	3795	31.75%	(30.92–32.59)
Residency	Urban	17 860	72.65%	5129	29.49%	(28.81–30.17)
	Rural	6724	27.35%	1861	28.41%	(27.33–29.51)
Age groups	15–24	3373	13.73%	928	28.10%	(26.60–29.70)
	25–44	9242	37.61%	2844	31.40%	(30.50–32.40)
	45–64	8528	34.71%	2350	28.30%	(27.40–29.30)
	≥ 65	3429	13.95%	867	26.30%	(24.80–27.80)
Marriage status	Married	17 948	73.36%	4992	28.52%	(27.85–29.19)
	Widowed	1306	5.34%	417	33.17%	(30.61–35.81)
	Divorced	313	1.28%	138	45.70%	(40.14–51.33)
	Single	4837	19.77%	1401	29.54%	(28.26–30.86)
	Separated	61	0.25%	26	44.07%	(31.94–56.77)
Education	Illiterate and Read &Write	4380	17.90%	1294	30.77%	(29.39–32.18)
	Elementary & secondary	5644	23.07%	1603	29.03%	(27.85–30.24)
	Diploma	6172	25.22%	1735	28.85%	(27.71–30.00)
	Above diploma & Bachelor	6454	26.38%	1841	29.08%	(27.97– 30.21)
	Master and above	1818	7.43%	500	27.87%	(25.83–29.98)
Job status	Employed	9860	40.32%	2605	27.05%	(26.17–27.94)
	Unemployed	1553	6.35%	586	38.86%	(36.42–41.34)
	Student	2071	8.47%	562	27.52%	(25.62–29.49)
	Housewife	7963	32.56%	2488	32.17%	(31.14–33.22)
	Retired & Pensioner	2794	11.43%	653	23.98%	(22.41–25.61)
	Unable to work	214	0.88%	74	35.75%	(29.45–42.44)
Total		23 945		6990	29.70%	(29.80–28.60)

 We applied the age-sex standardized prevalence based on a combination of the age-sex population of Iran. The standardized relative frequency distribution of the GHQ-28 subscales in the Iranian individuals aged 15 years and older showed that, in general, the highest percentage of suspected mental disorders was in the dimension of anxiety (32.34%), and the lowest percentage was in the dimension of depressive symptoms (25.24%). The prevalence of suspected mental disorders in all four domains of the GHQ-28, including anxiety, depression, social dysfunction, and somatization, was higher in women than men (Table 2).

**Table 2:** Standardized Relative Frequency Distribution of GHQ-28 Subscales in Population Over 15 Years of Age after COVID-19

**GHQ-28 Subscales**		**Male**	**Female**	**Total**
Somatization	Suspected of disorder (Score 2 and more)	23.49%	31.60%	27.52%
	Healthy (Score 1 and less)	76.51%	68.40%	72.48%
Anxiety	Suspected of disorder (Score 2 and more)	29.18%	35.55%	32.34%
	Healthy (Score 1 and less)	70.82%	64.45%	67.66%
Social dysfunction	Suspected of disorder (Score 2 and more)	28.00%	30.29%	29.13%
	Healthy (Score 1 and less)	72.68%	68.12%	70.41%
Depression	Suspected of disorder (Score 2 and more)	24.79%	25.70%	25.24%
	Healthy (Score 1 and less)	75.21%	74.30%	74.76%
Mental disorders	Suspected of disorder (Score 6 and more)	27.32%	31.88%	29.59%
	Healthy (Score 5 and less)	72.68%	68.12%	70.41%

 Comparison of the mean total score of the test and that of each GHQ-28 subscale in terms of gender showed a statistically significant difference (*P* = 0.001) such that the mean scores in all 4 dimensions and the total score of the test were higher in women than men (Table 3).

**Table 3:** Comparing the Mean Scores of the Participants’ Scores in GHQ-28

**GHQ-28 Subscales/Gender**		**Mean**	**Standard Deviation**	***t***	***df***	***P***
Somatization	Male	1.32	2.39	-11.40	24 390.66	0.001
	Female	1.67	2.51			
Anxiety	Male	1.53	2.44	-9.64	24 397.94	0.001
	Female	1.84	2.55			
Social dysfunction	Male	1.52	2.44	-4.01	24 353.56	0.001
	Female	1.65	2.53			
Depression	Male	1.35	2.45	-2.98	24 246.34	0.003
	Female	1.44	2.51			
Total	Male	5.71	9.28	-7.33	23 903.34	0.001
	Female	6.61	9.63			

 Logistic regression analysis showed that the OR of having symptoms of suspected mental disorders in women was greater than men (OR = 1.195, 95% CI 1.10–1.29). The OR of having suspected mental health symptoms in the 25–44 years age group was higher than those aged under 25 years (OR = 1.206, 95% CI 1.06–1.36). Also, factors such as place of residence, marital status, education level, and employment of individuals predicted mental health status such that the presence of symptoms of suspected mental disorders was higher in city residents (OR = 1.116, 95% CI 1.04–1.19), the illiterate (OR = 1.286, 95% CI 1.11–1.48), divorced (OR = 1.924, 95% CI 1.50–2.45), and unemployed (OR = 1.657, 95% CI 1.40–1.94) compared to the other groups (Table 4).

**Table 4:** Logistic Regression Analysis of Suspected Mental Disorders According to Demographic Factors

**Variables**		**Unadjusted**		Adjusted*		
		**OR**	**95% CI**	**OR**	**P value**	**95% CI**
Gender	Men (ref)					
	Female	0.781	(074, 0.83)	1.196	0.001	(1.10, 1.29)
Residency	Rural (ref)					
	Urban	1.054	(0.99, 1.12)	1.116	0.002	(1.04, 1.19)
Age group	15–24(ref)					
	25–44	1.172	(1.07, 1.28)	1.206	0.003	(1.06, 1.36)
	45–64	1.010	(0.92, 1.11)	1.011	0.870	(0.88, 1.15)
	≥ 65	0.911	(0.82, 1.01)	0.841	0.037	(0.71, 0.98)
Marital status	Single (ref)					
	Married	0951	(0.89, 1.02)	0.960	0.414	(0.87, 1.05)
	Widowed	1.184	(1.04, 1.35)	1.159	0.079	(0.98, 1.36)
	Divorced	2.007	(1.59, 2.54)	1.924	0.001	(1.50, 2.45)
	Separated	1.879	(1.12, 3.15)	1.805	0.027	(1.06, 3.04)
Education	Master and above (ref)					
	Above diploma & bachelor	1.150	(1.02, 1.30)	1.070	0.264	(0.95, 1.20)
	Diploma	1.059	(0.94, 1.19)	1.064	0.326	(0.94, 1.20)
	Elementary & secondary	1.049	(0.93, 1.18)	1.125	0.069	(0.99, 1.27)
	Illiterate and read & write	1.061	(0.94, 1.19)	1.286	0.001	(1.11, 1.48)
Job-status	Student (ref)					
	Employed & pensioner	0.976	(0.88, 1.09)	0.968	0.668	(0.83, 1.12)
	Unemployed	1.674	(1.45, 1.93)	1.657	0.001	(1.40, 1.94)
	Retired	1.465	(1.08, 1.98)	1.742	0.001	(1.25, 2.41)
	Unable to work	0.831	(0.73, 0.95)	0.952	0.589	(0.79, 1.13)

*The inter method at a 95% confidence level.

 In the present study, 14.7% (n = 3624) of individuals reported a history of COVID-19 infection. The majority of participants (51.7%) experienced the infection for 1 to 3 months (52.1%). Also, the percentage of COVID-19 infection or death due to this disease in their families was 32.3% and 13.2%, respectively.

 Suspected mental disorders had a statistically significant association with the COVID-19 infection status, the experience of infection or death due to COVID-19 in their families, severity, and duration of COVID-19 infection: suspected mental disorders were found in 40%, 35.9%, and 39.6% of individuals with a history of infection, the experience of infection in their families, and bereaved families, respectively (*P* < 0.05). Furthermore, the rate of suspected mental disorders increased with increasing severity of COVID-19 infection and decreased when passed more than one month since the infection (Table 5).

**Table 5:** Distribution of COVID-19 Infection in Individuals and Their Family Concerning Suspected Mental Disorders

**Variable**		**Sample Size**		**GHQ≥6**		**GHQ<6**		***P***
		**No**.	**%**	**No**.	**%**	**No**.	**%**	
Infected person by COVID-19	Yes	3624	14.7	1411	40	2117	60	0.001
	No	20958	85.3	5579	27.3	14837	72.7	
Intensity of the infection	Mild	1868	51.7	646	35.5	1176	64.5	0.001
	Moderate	1099	30.4	449	42	621	58	
	Severe	646	17.9	314	50.2	312	49.8	
Time passed since the infection (month)	< 1	128	3.6	66	54.11	56	45.9	0.001
	1–3	1863	52.1	738	40.5	1085	59.5	
	4–6	866	24.2	312	37.2	526	62.8	
	7–9	307	8.6	121	40.7	**176**	59.3	
	10–12	409	11.4	156	38.9	**245**	61.1	
Family infected by COVID-19	Yes	7928	32.3	2775	35.9	4954	64.1	0.001
	No	16643	67.7	4211	26	11995	74	
Family death due to COVID-19	Yes	1050	13.2	404	39.6	615	60.4	0.008
	No	6884	86.8	2374	35.3	4342	64.7	

## Discussion

 The present research was a population-based study conducted aiming to determine the mental health status of individuals over 15 years of age in the Iranian population. The results of the current research showed that the overall prevalence of suspected mental disorders was 29.7% in the population over 15 years of age one year after the outbreak of the COVID-19 epidemic in Iran; also, the results showed that the overall prevalence of suspected mental disorders in women, city residents, the age group 25 to 44 years, divorced, illiterate, and unemployed individuals, people with a history of COVID-19 disease, and those who experienced the infection or death of relatives due to COVID-19 was higher than others. Similarly, the rate of suspected mental disorders increased with increasing severity of COVID-19 disease and decreased when passed more than one month since the infection. Numerous studies have been conducted for many years on mental health status in different groups of the Iranian society. The first national study of mental health assessment in the Iranian population over 15 years of age was conducted by Noorbala et al. The prevalence of suspected mental disorders using the GHQ-28 was 21%.^9^ In the second national study carried out by Mohammadi et al on mental health assessment using the Schedule for Affective Disorders and Schizophrenia (2001), it was shown that 17.10% of Iranians over 18 years of age suffered from at least one psychiatric disorder.^18^ The third national study was conducted by Sharifi et al on the epidemiology of psychiatric disorders in Iran using the Composite International Diagnostic Interview-Second Edition (CIDI-2) and the GHQ-28. In their study, they reported that 23.6% of individuals 15–64 years of age had suffered from at least one psychiatric disorder during the past 12 months, accounting for 26.5% in women and 20.8% in men.^19^ The fourth national study was conducted by Noorbala et al on mental health in Iran using the GHQ-28. The results showed that a total of 23.44% of the subjects were suspected of having a mental disorder.^10^ The prevalence of suspected mental disorders in the present study was not consistent with the results of the above-mentioned studies. The reason for this inconsistency can be due to differences in the statistical population and different types of methodology and measurements. Considering these differences, this finding shows that the prevalence of mental disorders has been increasing over the years in Iran, and the incidence of COVID-19 and the special post-COVID-19 conditions can impose an additional burden in this regard. Furthermore, we experience severe economic pressures (most of them unrelated to the COVID-19) in recent years in Iran.

 According to the results of a meta-analysis published in 2020, the prevalence of psychiatric disorders in Iran based on screening tests and clinical interviews was 31.03% and 25.42%, respectively,^7^ almost consistent with the results of the present study. In another study performed to examine the longitudinal changes in mental health status from 1999 to 2015 in the Iranian population over 15 years of age, the prevalence of suspected mental disorders was shown to shift from 21% in 1999 to 23.4% in 2015. Also, in over 15 years, the prevalence of suspected mental disorders has increased 1.12 times. The frequency distribution of suspected mental disorders in terms of place of residence in 1999 changed from rural to urban population in 2015. However, in both periods, the prevalence of mental disorders in individuals over 65 years of age, as well as divorced and illiterate individuals was higher than other groups.^20^ The high prevalence of mental disorders is not only related to Iran but is also a global concern such that according to the results of reviewing 42 articles published between 1978 and 2015, the probability of the occurrence of mental disorders has increased 1.17 times in most countries.^21^

 The results of the current research were different from the results of Noorbala et al in terms of the general prevalence of suspected mental disorders and their distribution in age groups; in the present study, individuals aged 25 to 44 years had the highest percentage of disorders, showing that the frequency of disorders has shifted to the young-to-middle age group compared to 2015. The overall prevalence of suspected mental disorders has also increased compared to 2015. However, similar to previous years, the individuals’ place of residence, literacy level, and marital status are factors associated with the prevalence of suspected mental disorders. The observed difference may be due to the prevalence of the COVID-19 epidemic and rapid demographic, social, and economic changes in Iran, which play an important role in shaping the individuals’ health and might sound the alarm for the officials and planners in the field of mental health so that, with proper planning, the resultant adverse consequences can be prevented in this regard.

 In the present study, the factors of age, gender, place of residence, marital status, education level, and employment predicted mental health status such that the presence of symptoms of suspected mental disorders in city residents, and illiterate, divorced, and unemployed individuals was more frequent than other groups.

 In a similar study in the form of a national mental health survey, Tavousi et al assessed the mental health of 19 949 individuals of the Iranian population using the GHQ-28. The reported results showed that 29.8% of the population aged 18 to 65 years had some degree of mental disorders. Also, mental disorders were more common in women than men, but the difference was not statistically significant, while the relationship of mental health status with increasing age, education, and unemployment was statistically significant.^22^ The results of the present study were consistent with the results of the above-mentioned studies in terms of the overall prevalence of suspected mental disorders but not in terms of some predictors of mental health, including education level and age. The observed difference can be due to differences in the age range of the statistical population, as well as the scoring method of mental health measuring instruments compared to the above-mentioned studies. This finding indicates that environmental factors are as much involved in the incidence of symptoms of suspected mental disorders as biological factors. However, most environmental factors are modifiable or preventable.

 The results of the present study showed that the highest and the lowest percentage of suspected mental disorders pertained to the anxiety subscale (32.40%) and the scope of depressive symptoms (24.80%), respectively. The prevalence of suspected mental disorders in all four domains of the GHQ-28, including anxiety, depression, social dysfunction, and somatization, was higher in women than men. Also, the assessment of the status of COVID-19 infection in the participants showed that 14.7% (n = 3,624) reported a history of COVID-19 infection.

 The prevalence of suspected symptoms of anxiety disorder and depression in the present study was higher than the findings of Shahriarirad et al who reported that during the outbreak of COVID-19, 15.1% and 20.1% of the Iranian general population over 15 years of age had experienced clinical symptoms of depression and anxiety, respectively. Higher education had been reported as a protective factor, and the female gender and the high number of members in a family had been reported as risk factors.^16^ The observed differences may be due to the psychological effects associated with the prolongation of the COVID-19 epidemic.

 Similar to Iran, the COVID-19 epidemic has had unprecedented effects on the mental health of people in other parts of the world. The results of the systematic review showed that during the COVID-19 epidemic, the general population of some countries, including Denmark, China, Italy, Nepal, and Iran, experienced anxiety symptoms from 6.33% to 50.9% and depressive symptoms from 14.6% to 48.3%. Female gender, younger age group (40 years of age), the presence of chronic mental illnesses, unemployment, and student status were the most important risk factors associated with the infection.^23^ In a retrospective study in the United States, it was found that 18.1% of the individuals who recovered from COVID-19 had one of the symptoms of a psychiatric disorder 14 to 90 days after the infection, 5.8% of whom were new cases. In individuals over 65 years of age, the prevalence of dementia increased by 1.6%.^14^ A similar result has been also reported in the United Kingdom regarding the prevalence of mental disorders following an increase in the COVID-19 pandemic.^24^ The results of the mentioned studies are higher than the results of our study in terms of anxiety disorder and depression. The observed difference can be due to differences in the methodologies of the studies or cultural-social differences.

 As a psychological and social phenomenon, mental health plays a role not only in the mental quality of individuals but also in creating a healthy and positive life for all members of the society and consequently, a healthy social environment. The prevalence of mental disorders is a social emergency and requires proper planning and intervention that should be considered by planners and policymakers. The increasing prevalence of mental disorders in our results compared to 2015 shows that the planning and policy-making may not have been sufficient to reduce mental disorders in previous years or effective in critical situations such as the COVID-19 epidemic.

###  Limitations

 One of the limitations of the present study was the lack of necessary conditions for conducting a face-to-face survey due to the prevalence of COVID-19. Therefore, to overcome this limitation, by observing scientific and precise instructions, the telephone data collection method was used. The second limitation was the self-reporting method of data collection. Therefore, the reported rate of infected COVID-19 cases may be different from the official reports. It is suggested that the findings of the present study should be used and generalized considering the above-mentioned limitation.

## Conclusion

 In conclusion,the results of the present study showed that the mental health status of the population over 15 years of age in Iran had an increasing trend compared to 2015. Also, people with COVID-19 and bereaved families had a higher prevalence of mental health disorders. The observed difference may be due to the prevalence of the COVID-19 epidemic and rapid demographic, social, and economic changes in Iran. The prevalence of disorders in terms of subscales showed that anxiety disorder was reported higher than social dysfunction and somatization. Planning to improve mental health in the Iranian population should be considered for the post COVID-19 era, like other parts of the world, especially in women, low-educated and unemployed individuals.

## References

[1] Noorbala AA, Bagheri Yazdi SA, Asadi Lari M, Vaez Mahdavi MR (2011). Mental health status of individuals fifteen years and older in Tehran-Iran (2009). Iran J Psychiatry Clin Psychol.

[2] Damari B, Almadani SH, Hajebi A, Salehi Shahrabi N (2019). Promoting mental health in workplaces of Iran; reviewing present status and future approaches. Iran J Psychiatry Clin Psychol.

[3] World Health Organization. Mental Health Atlas. Switzerland: World Health Organization, 2005.

[4] Vos T, Allen C, Arora M, Barber RM, Bhutta ZA, Brown A (2016). Global, regional, and national incidence, prevalence, and years lived with disability for 310 diseases and injuries, 1990-2015: a systematic analysis for the Global Burden of Disease Study 2015. Lancet.

[5] World Health Organization (‎WHO)‎. mhGAP: Mental Health Gap Action Programme: Scaling up Care for Mental, Neurological and Substance use Disorders. Geneva: WHO; 2008. 26290926

[6] Gustavson K, Knudsen AK, Nesvåg R, Knudsen GP, Vollset SE, Reichborn-Kjennerud T (2018). Prevalence and stability of mental disorders among young adults: findings from a longitudinal study. BMC Psychiatry.

[7] Taheri Mirghaed M, Abolghasem Gorji H, Panahi S (2020). Prevalence of psychiatric disorders in Iran: a systematic review and meta-analysis. Int J Prev Med.

[8] Moradpour F, Hajebi A, Salehi M, Solaymani-Dodaran M, Rahimi-Movaghar A, Sharifi V (2019). Province-level prevalence of psychiatric disorders: application of small-area methodology to the Iranian Mental Health Survey (IranMHS). Iran J Psychiatry.

[9] Noorbala AA, Bagheri Yazdi SA, Yasamy MT, Mohammad K (2004). Mental health survey of the adult population in Iran. Br J Psychiatry.

[10] Noorbala AA, Faghihzadeh S, Kamali K, Bagheri Yazdi SA, Hajebi A, Mousavi MT (2017). mental health survey of the Iranian adult population in 2015. Arch Iran Med.

[11] Wang C, Pan R, Wan X, Tan Y, Xu L, Ho CS, et al. Immediate psychological responses and associated factors during the initial stage of the 2019 coronavirus disease (COVID-19) epidemic among the general population in China. Int J Environ Res Public Health 2020;17(5). 10.3390/ijerph17051729 PMC7084952 32155789

[12] Balouch V, Vatanparast M, Dadpisheh S, Mirkazehi Rigi Z (2021). Stress, anxiety and depression status in the population of southern Sistan and Baluchestan province (coastal area) in the COVID-19 epidemic in 2020. J Mar Med.

[13] Daly M, Robinson E (2021). Psychological distress and adaptation to the COVID-19 crisis in the United States. J Psychiatr Res.

[14] Taquet M, Luciano S, Geddes JR, Harrison PJ (2021). Bidirectional associations between COVID-19 and psychiatric disorder: retrospective cohort studies of 62 354 COVID-19 cases in the USA. Lancet Psychiatry.

[15] Huang C, Huang L, Wang Y, Li X, Ren L, Gu X (2021). 6-month consequences of COVID-19 in patients discharged from hospital: a cohort study. Lancet.

[16] Shahriarirad R, Erfani A, Ranjbar K, Bazrafshan A, Mirahmadizadeh A (2021). The mental health impact of COVID-19 outbreak: a Nationwide Survey in Iran. Int J Ment Health Syst.

[17] Noorbala AA, Bagheri Yazdi SA, Mohammad K. The validation of general health questionnaire-28 as a psychiatric screening tool. Hakim Res J 2008;11(4):47-53. [Persian].

[18] Mohammadi MR, Davidian H, Noorbala AA, Malekafzali H, Naghavi HR, Pouretemad HR (2005). An epidemiological survey of psychiatric disorders in Iran. Clin Pract Epidemiol Ment Health.

[19] Sharifi V, Amin-Esmaeili M, Hajebi A, Motevalian A, Radgoodarzi R, Hefazi M (2015). Twelve-month prevalence and correlates of psychiatric disorders in Iran: the Iranian Mental Health Survey, 2011. Arch Iran Med.

[20] Noorbala AA, Bagheri Yazdi SA, Faghihzadeh S, Kamali K, Faghihzadeh E, Hajebi A (2017). Trends of mental health status in Iranian population aged 15 and above between 1999 and 2015. Arch Iran Med.

[21] Richter D, Wall A, Bruen A, Whittington R (2019). Is the global prevalence rate of adult mental illness increasing? Systematic review and meta-analysis. Acta Psychiatr Scand.

[22] Tavousi M, Haeri Mehrizi AA, Hashemi A, Naghizadeh F, Montazeri A. Mental health in Iran: a nationwide cross sectional study. Payesh 2016;15(3):233-9. [Persian].

[23] Xiong J, Lipsitz O, Nasri F, Lui LMW, Gill H, Phan L (2020). Impact of COVID-19 pandemic on mental health in the general population: A systematic review. J Affect Disord.

[24] Pierce M, Hope H, Ford T, Hatch S, Hotopf M, John A (2020). Mental health before and during the COVID-19 pandemic: a longitudinal probability sample survey of the UK population. Lancet Psychiatry.

